# Precision Nutrition Initiative: Toward Personalized Diet Recommendations for Patients With Inflammatory Bowel Diseases

**DOI:** 10.1093/crocol/otaa087

**Published:** 2020-10-19

**Authors:** Andrés Hurtado-Lorenzo, Gerard Honig, Caren Heller

**Affiliations:** Research Department, Crohn’s & Colitis Foundation, New York, New York, USA

There is a longstanding open question regarding how patients with inflammatory bowel diseases can best optimize their diet to maintain overall health, avoid triggering of symptoms, and support remission. However, to date there is limited evidence in support of comprehensive dietary recommendations for these patients. Experience with highly restrictive elimination diets indicates that diet-based therapy can be as effective as first-line medical therapy for many patients; however, such diets cannot be maintained over the lifetime. A new approach is needed to enable patients to maintain healthy and effective diets through less restrictive nutrition plans. The major challenge to date has been the variable individual response to specific foods; for one patient, a particular food may trigger symptoms while for another patient, that same food may be inert or even therapeutic. The process of determining which foods would be best for which patients remains based in trial and error at present due to the lack of biomarkers to predict or measure individual biological responses to foods. Recent technological advances enable a precision nutrition approach, in which personalized, optimized diets are be tailored to the biological, lifestyle, and clinical characteristics. Here, we describe a framework to advance precision nutrition for inflammatory bowel diseases though 4 synergistic approaches: clinical trials of evidence-based anti-inflammatory diets; identification of biomarkers to predict or measure responses to different diets; identification of dietary triggers of disease; and development of analytical tools based on artificial intelligence to predict response to food. We believe the time is right to create the evidence base and tools necessary to develop diets tailored to the clinical, lifestyle, and biological characteristics of patients as a novel and effective approach to improve disease outcomes.

## CHALLENGE AND OPPORTUNITIES

“What should I eat?” is one of the most common questions patients with inflammatory bowel diseases (IBDs) ask their doctors. Yet there is not a conclusive answer to this question and dietary recommendations for IBD patients remain based largely on trial and error for each individual patient.^1^

The goals for any nutrition plan for IBD are typically to provide a healthy diet while preventing exacerbation of symptoms and/or disease relapse once medical treatment has induced clinical remission.^2^ The immediate challenge to this seemingly simple objective is that patients can respond differently to the same food. While a certain food can be a trigger in a given patient, in other patients the same food can be therapeutic, leading to symptom improvement or relapse prevention. This is in part due to the fact that IBD is a highly heterogeneous disease with different underlying biology across patients^3^ leading to different responses to foods. An emerging concept and potential solution is precision nutrition, in which the nutritional plan is tailored to the biological, lifestyle, and clinical characteristics of each patient to deliver an optimal, personalized diet based on measurement and/or prediction of individual responses to foods.^4–6^

Advancing the ability to measure the personal response to food, while still in its infancy, has been enabled by rapid technological innovations in multiomics platforms for data generation and analysis, including nutrigenomics, microbial metagenomics, metabolomics, and proteomics.^4,7,8^ In particular, recent discoveries have elucidated relationships between dietary intake, human gut microbiome composition, microbial metabolite production, mucosal homeostasis, and immune responses.^9^ This creates a tremendous opportunity for data-driven research toward understanding how to measure personal responses to food and how to implement personalized dietary management of IBD.^5,6^ Moreover, the emerging field of nutrigenetics and nutrigenomics—defined as the study of the effect of genetic variation on dietary response and the role of nutrients and bioactive food compounds in gene expression^10,11^—supports the concept that variations in the human genome can influence the impact of food on the microbiome, immune response, and mucosal homeostasis.^12,13^ It is important to note that in addition to genotype variation and gene expression studies, nutrient–gene interactions can be further understood by the integration of epigenomics analysis. Diet on its own or by interaction with other environmental factors can cause epigenetic changes, leading to alterations in gene expression patterns.^14,15^ It has been shown that dietary molecules can act as epigenetic modulators of (1) DNA methylation, (2) histone acetylation/deacetylation, and (3) small noncoding RNA expression. Thus, dietary molecules can induce epigenetic changes by direct interaction with the enzymes responsible for adding or removing epigenetic marks or, indirectly, on the regulation of the expression of genes that encode proteins involved in the function of the epigenetic machinery.^14^

It is worth noting that a patient’s response to diet is a bidirectional activity, so that researchers should evaluate the effects of food on a given biological variable (eg, microbiome) and also the effect of that variable on the metabolism of food.^16^

In addition to the measurement of personal responses to diet based on biological factors, an important environmental variable to take into consideration when measuring personal response to food is the variation in lifestyle, in particular in patterns of physical activity (PA). Variation in the levels of PA may affect individual metabolism and thus the response to diet.^16^ In addition, PA may also have an influence on genetic predisposition to metabolic disturbances. For instance, it has been reported that sedentary behavior correlates with increased body mass index (BMI) in carriers of BMI-predisposing polymorphisms whereas greater PA attenuated the genetic effects on BMI.^17^ The current availability of accelerometers integrated in wearable devices will make the analysis of PA a more objective measure (compared to self-report questionnaires) that can be incorporated into precision nutrition studies.^18,19^

Overall, precision nutrition research in IBD should thus focus on the identification of biological parameters that reflect and/or predict IBD patient’s physiological response to foods, based on the analysis and integration of 1 or more sources of patient data (such as nutrigenomics, epigenomics, microbiomics, metabolomics, and/or proteomics), together with tracking of food consumption, PA^4,8^ and relevant patient outcomes (eg, remission, relapse, exacerbation).^2^ These biomarker signatures should predict patient-level responses to specific foods, including triggering of disease (eg, symptom exacerbation) and may identify subgroups of patients likely to respond well to a specific therapeutic diet leading to symptom improvement and disease remission.^4^ Ideally, identified signatures should also provide a source of hypotheses to implement studies aimed to understand the exact mechanism of action (MoA) of foods with beneficial or deleterious effects in response to the unique biology of each patient, their diets, or individual food components.

In addition to the implementation of multiomics platforms to facilitate the advancement of precision nutrition, there is also potential for biosensor and biosampling technologies. In particular, novel biosensor technologies integrated into swallowable capsules^20^ or wearable bracelets^21^ are in development for continuous monitoring of intestinal inflammatory markers, which could be adapted for the identification, in real time, of foods capable of inducing inflammation. In addition, gas sensor pills, which take longitudinal measurements of gas profiles in the gut can be correlated to intestinal transit times as indirect measurement, for instance, of the effect of high vs low fiber diet.^22^ Gas profiles, which are modified by diet,^23^ may also relate directly to specific metabolic and digestive processes and/or to specific pathophysiological differences in nutrient metabolism.^24,25^ Finally, “pill samplers”—swallowable capsules that collect luminal fluids and microbes along the gastrointestinal—may be useful to measure nutrients as well as metabolite and microbiome composition allowing a precise characterization of differential diet–microbiota–host interactions along different areas of the GI tract.^23,26^

Thus taken together, biosensor/biosampler generated information and multiomic derived biomarkers will represent an unprecedented approach in defining the mechanism by which nutrition plays a role in heath and disease, and should support evidence-based design of personalized anti-inflammatory diets for patients, in order to prevent disease relapses and exacerbation of symptoms or induce remission.

Recognizing the importance and need to elucidate the impact of individual diets on health and disease, the National Institutes of Health launched the *2020–2030 Strategic Plan for NIH Nutrition Research* to create a research framework to advance the field of precision nutrition.^27^

## PRECISION NUTRITION CLINICAL PROOF OF CONCEPT

Research in the field of precision nutrition has evolved from the pioneering work of Segal and Elinav and colleagues.^28,29^ In one important study, glucose levels were continuously monitored, and meal compositions were recorded, revealing that the impact of a particular food on blood glucose was highly variable from individual to individual and suggesting that personalized, rather than universal dietary recommendations were needed for optimal glycemic control. A range of physiological and microbiome parameters were also assessed and evaluated using machine learning to develop a predictive model, incorporating blood markers, dietary habits, PA, and microbiome features, able to predict individual glycemic responses to specific foods and then make personalized dietary recommendations. Personalized diet plans were then compared to standard dietary recommendations and were shown to improve glycemic response to a dietary challenge.^28^ The model was further evaluated in a US validation cohort study.^30^

More recently, the PREDICT 1 study evaluated postprandial metabolic responses to food, including glucose tolerance, insulin sensitivity, and additional metabolic parameters, in a large real-world cohort, including twin pairs. Comprehensive assessment of individual responses to meals was performed, taking into account meal composition, blood markers, microbiome markers, circadian PA, and meal timing to develop machine learning based predictive models for individual responses to foods. The models were also shown to be predictive of risk of metabolic syndrome and cardiovascular disease as assessed using widely accepted risk assessment methods.^19,31^ Taken together, these results provide proof of concept that a precision nutrition approach is feasible and sets the stage for its application in different therapeutic areas, including IBD.

## CROHN’S & COLITIS FOUNDATION PRECISION NUTRITION INITIATIVE FOR IBD

The application of the precision nutrition paradigm in the IBD field is an emerging concept. Focused studies that evaluate individual responses to food based on the analysis of well-defined biological factors and their correlation with the improvement of clinical endpoints in IBD are still lacking. Addressing this important patient need, the Crohn’s & Colitis Foundation (Foundation) has identified the goal of understanding how diet affects IBD, particularly at the individual patient level, as a critical gap in the understanding and management of these diseases, and as an opportunity to make a significant impact on the quality of life of patients. In order to address this knowledge gap and provide a solid and focused platform to advance this field, the Foundation has recently launched a precision nutrition research initiative. The overarching goal of this initiative is to develop methodologies that will enable measuring and incorporating individual biological, clinical, and lifestyle characteristics of a patient, together with the mechanistic understanding of specific food effects on disease outcomes, to design comprehensive, personalized nutrition plans for IBD patients.

The expectation is that this knowledge would provide an evidence-based rationale for randomized control trials (RCTs) to evaluate the efficacy of rationally designed therapeutic diets in improving patient outcomes. The long-term goal would be to generate predictive models, including quantitative and qualitative measurement of responses to food, which could be integrated into the discussion between patients and practitioners about personalized IBD management. Importantly, there is strong evidence that dietary interventions can in fact be as effective as medical therapy for the management of inflammation in certain IBD populations. Notably, exclusive enteral nutrition (EEN), a highly restrictive elimination diet, has been adopted as a first-line therapy to induce remission in pediatric Crohn disease (CD) in many countries; its efficacy (comparable to steroids) suggests that elimination of dietary triggers of inflammation can be highly beneficial, safe, and cost-effective.^32–35^ However, EEN is difficult to maintain over the long term and does not allow patients to enjoy the health and lifestyle benefits of a varied diet. Therefore, more personalized and less restrictive diets, in which a smaller number of dietary triggers specific to the individual patient’s physiology would be eliminated, would be highly attractive to patients.

Research into dietary management of IBD has historically not received a level of investment comparable to research into pharmacological interventions^1^; however, it is important to recognize that the impact of diet is of great interest to IBD patients. IBD Partners is a patient-centric research program, funded by the Foundation and led by the University of North Carolina School of Medicine. Diet has been consistently ranked by patients and caregivers through the IBD Partners crowdsourcing and voting process as one of the most critical research priorities in the field.

If precision nutrition is to become a reality, research must focus on identifying biological signatures and/or mechanisms of response to food in IBD patients and their correlation with disease outcomes. At a minimum, the following is needed: (1) Identification of associations between baseline features (eg, microbiome composition) and response to diet (eg, clinical remission or relapse); (2) Testing of these associations prospectively in a population to determine whether predicted responses are correct; and (3) Evaluation of whether personalized recommendations based on predictions result in better outcomes.^16^

Advancing precision nutrition also depends on clinical studies of ethnically diverse and appropriately sized cohorts of patients with well-defined phenotypes, accurate tracking of environmental exposures and harmonized processes to enable comparison of data across studies. Considerations for IBD cohort studies of environmental factors, including diet, have been reviewed in detail by the Foundation’s Environmental Triggers working group.^36^ In addition, we have recently reviewed the needs and challenges related to harmonization in biomarker research practices^36^; the Foundation’s IBD Plexus initiative is an example of an effort designed to create harmonized cohort-based data in the IBD field.^37,38^

In order to advance the field of precision nutrition in IBD, the Foundation has designed a research portfolio that integrates the expertise of 4 research teams from 3 different countries, United States, Canada, and Scotland. Each team represents a multidisciplinary group that includes nutrition research specialists, IBD clinicians and researchers, molecular biologists, immunologists, and bioinformaticians.

**FIGURE 1.**
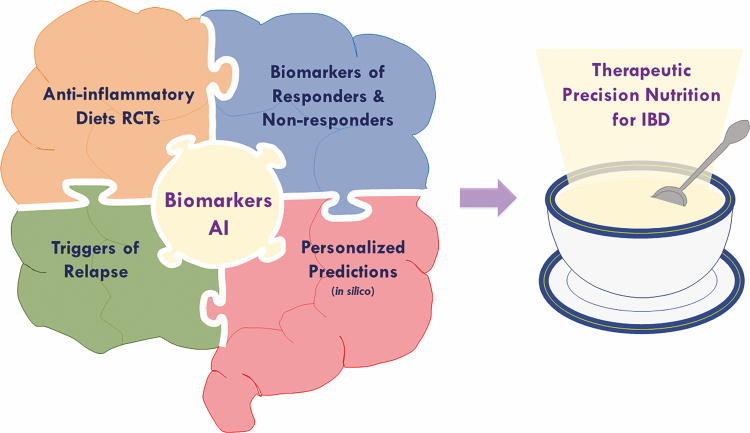
Key components of the Foundation’s precision nutrition initiative for IBD. Driven by a multi-institutional approach, this initiative integrates several key elements needed to assemble the IBD precision nutrition puzzle, with the overarching goal of providing unified scientific evidence to support the implementation of personalized diet recommendations for the management of IBD. These key elements are: (1) evaluation of rationally designed anti-inflammatory diets to improve disease outcomes in randomized clinical trials, (2) identification of biomarkers to predict subgroups of IBD patients who are responders and nonresponders to these anti-inflammatory diets, (3) identification of proinflammatory “trigger foods” that can exacerbate symptoms or induce disease relapse, and (4) development of in silico predictive tools required to design evidence-based therapeutic diets, whose efficacy can be evaluated in trials. A critical unifying element at the core of this initiative is the use of artificial intelligence (AI)-based analysis of multiomics data for the identification of the biomarkers required to make predictions of patients’ response to food, in order to enable precise and effective therapeutic diet recommendations.

The Foundation’s precision nutrition research portfolio is crafted in a way that the whole is more than the sum of its parts. Each research project provides a unique approach that will contribute an important piece of information to help assemble the precision nutrition puzzle (Fig. 1), leading to a clearer vision of how diet relates to the clinical presentation and management of IBD. More importantly, they will provide scientific evidence to understand how patients respond to food and how to tailor diet to the biological and clinical characteristics of patients based on the identification of multiomics-based biomarkers and predictive analytical tools, to support the potential use of diet as personalized therapy for IBD. As shown in Figure 1, these projects are focused on 4 main topics:

1) Evaluation of evidence-based and rationally designed anti-inflammatory diets in RCTs.
2) Identification of biomarkers to predict and/or assess responders and nonresponders to these different diets.
3) Identification of dietary triggers of relapse.
4) Development of in silico analytical tools to predict treatment response to food leading to rational design of personalized dietary recommendations.

Each project addresses the challenge of precision nutrition in IBD in a unique manner, as outlined below.

1) *Whole food diet RCT for CD*: Based on 3 lines of experimental evidence supporting: (1) the beneficial effects of certain bacterial metabolites on amelioration of inflammation,^39–41^ (2) the effects of dietary phytonutrients on increasing beneficial intestinal microbial taxa^42^ and on suppression of IBD-associated microbes (eg, Escherichia *coli*),^43–46^ and (3) the effects of phytonutrients on the induction of anti-inflammatory bacterial metabolites,^47–50^ Dr Li and collaborators from the University of California, Los Angeles (UCLA) have designed an anti-inflammatory whole food diet based on the exclusion of proinflammatory foods plus the addition of anti-inflammatory phytonutrients and prebiotics. The goal of this study is to evaluate the efficacy of this whole food diet on induction of remission in CD in a RCT. The primary outcome will be clinical response at week 8. Secondary outcomes will include clinical remission, reduction in fecal calprotectin and lipocalin 2, and changes in patient-reported measures of PA, mood, fatigue, pain, sleep, and quality of life. Serum cytokine and C-reactive protein assays will be performed to assess the inflammatory response. This study will identify clinical, PA, and multiomic signatures based on microbiomics, metabolomics, and microRNA,^51–53^ to stratify responders and nonresponders, which is the core of precision nutrition.
2) *Diet to optimize fatty acid profile and nutrient composition in CD*: In order to design diets that can support long-term health in IBD patients, it is important to consider that many patients are chronically malnourished, presumably due to individual differences in diet (eg, avoidance of many foods as potential triggers) and/or differences in absorption of specific nutrients.^42^ This issue illustrates the need for more precise dietary recommendations taking into account not only avoidance of specific dietary triggers, but also the overall nutritional needs of the patient. Drs Raman and colleagues at the University of Calgary are conducting a clinical trial to assess whether a whole foods anti-inflammatory diet, designed using a conceptually related approach as employed by Li and colleagues (Project #1), but also optimized to ensure intake of polyunsaturated fatty acids. The goal of the intervention is to induce steroid-free remission in patients with mild-to-moderate CD. The diet is designed and supplemented to maximize intake of specific nutrients previously shown to be deficient in many patients with CD,^42^ and dietary caloric intake is personalized according to the patient’s nutritional status at baseline. The primary outcomes are clinical remission and fecal calprotectin. In addition, multiple physiological markers will be assessed using ‘omics and ex vivo systems, including host lipidomics, which will be particularly important to characterize metabolism of fatty acids. Additional research endpoints include metabolomics, microbiome composition, and function of immune cells. The goal of the ‘omics analyses is to identify biomarkers to predict therapeutic response to diet in CD. Importantly, this study incorporates the use of wearable inclinometers and accelerometers to track and incorporate PA and sedentary time into predictive models.
3) *Identification of dietary triggers of relapse*: In order to advance the precision nutrition field, the identification of proinflammatory dietary components that can trigger symptoms exacerbation and relapse, as well as elucidation of the underlying patient-specific biological factors, are critically important. Prof. Gerasimidis and colleagues at the University of Glasgow, the University of Edinburgh, the University of Strathclyde, the Royal Hospital for Children in Glasgow, and the Royal Hospital for Sick Children will identify dietary triggers of relapse leveraging the observation that in the clinic EEN induces clinical remission in 80% of children with active CD^32–34^ and that in most patients this therapeutic effect is lost within 17 days of introducing a normal diet despite the use of immunosuppressants or partial enteral nutrition.^54^ These observations suggest that dietary components overshadow the benefits of EEN treatment, and offers an excellent experimental clinical model to identify proinflammatory foods capable of triggering disease relapse. This elegant research approach profits from the critical window of reactivation of gut inflammation following completion of EEN and return to habitual diet to identify dietary triggers of inflammation. EEN alters microbiome and metabolome profiles suggesting that the therapeutic effects of EEN are due in part to these changes.^55–57^ Thus, the effects of identified dietary triggers of inflammation on intestinal microbiome, metabolome, and immunological profiles will be evaluated in addition to clinical parameters of disease activity. Collectively, this study will advance the understanding of the mechanisms by which food modifies disease activity and initiates gut inflammation, and will identify the biological determinants underlying response to different diets of CD patients in their daily life. In addition to dietary triggers identification, a secondary goal of this study is to evaluate the efficacy of the CD-TREAT diet within a RCT. CD-TREAT is a nutritionally complete diet, designed to reproduce the nutritional composition of EEN.^58^ CD-TREAT contains ordinary foods, with the exclusion of certain dietary compounds (eg, lactose and gluten), to resemble nutrient exclusions of EEN.^58^ The aim is therefore to use CD-TREAT to prevent or reduce the induction of intestinal inflammation during food introduction post-EEN intervention as a potential dietary strategy for sustained maintenance of remission.
4) *In silico personalized predictions of response to diet*: While new prospective diet studies in IBD are critical, it is also important to leverage and integrate data across studies and cohorts to define relationships between specific food components, metabolism and outcomes in specific patient populations. Such integration requires the development and implementation of novel analytical approaches including high-throughput metabolomics and artificial intelligence. Drs Dorrestein and Knight and colleagues at the University of California, San Diego (UCSD) are taking advantage of multiple technological advances in order to pursue this goal. Using large data sets representing the molecular components of many real-world foods,^59^ metabolomics from individuals who have consumed those foods, and processing of dietary molecules by the microbiome,^60^ the investigators are applying computational methods (source tracking and molecular networking^61^) to derive the molecular composition of an individual’s diet based solely on metabolomics and microbiome analyses. Machine learning approaches will then be used to identify associations between food components, microbiome composition, and clinical outcomes in historical IBD data sets and to rationally design diets based on these predictions.

This approach is enabled by recent technical advances and data set availability and illustrates the potential for artificial intelligence methods to integrate real-world data and clinical trial outcomes to advance precision nutrition for IBD. Importantly, one of the most challenging aspects of this approach is the need to convert molecular and clinical data into a uniform format suitable for integrated analysis. Improved harmonization of data collection and reporting in IBD studies could greatly accelerate such integrative analyses across cohorts, as we have previously recommended.^37^

## CONCLUSIONS

Answering a recurrent question raised by IBD patients—“what should I eat to manage my disease?”—remains based largely on a trial-and-error process at present. Clinicians lack sufficient scientific evidence and predictive tools to deliver the right diet to the right patient at the right time in order to ameliorate symptoms, induce remission, and prevent disease relapse. To address this unmet need, the emerging concept of precision nutrition provides a framework in which the integration of clinical, lifestyle, and biological parameters is used to predict individual patient responses to food, enabling the design of personalized and efficacious dietary plans to help manage disease.

To advance the field of precision nutrition in IBD, the Foundation launched a pioneering multi-institutional effort, that integrates: (1) the evaluation of anti-inflammatory diets to improve disease outcomes in RCTs, (2) the development of predictive models and biomarkers to identify subgroups of IBD patients who are responders and nonresponders to these diets, (3) the identification of proinflammatory “trigger foods” that can exacerbate symptoms or induce disease relapse in certain patients, and (4) development of artificial intelligence based in silico predictive tools required to rationally design therapeutic diets whose efficacy can subsequently be evaluated in RCTs.

We are confident that the time is right, with the technological and analytical tools now sufficiently advanced, to create the evidence base and predictive tools necessary to increase the confidence and the armamentarium of clinicians, to support the concept that diets tailored to the clinical, lifestyle, and biological characteristics of IBD patients, may represent a novel and effective approach to improve disease outcomes.

## Data Availability

Data sharing is not applicable to this article as no new data were created or analyzed in this study.

## Notes

Funding: The precision nutrition initiative is sponsored by generous donations from Jonathan D. Rose, MD, PhD, Chair of the Crohn’s & Colitis Foundation’s Intestinal Pathology Research Program and UnitedHealth Group.
